# Microglia-Specific Expression of *Olfml3* Is Directly Regulated by Transforming Growth Factor β1-Induced Smad2 Signaling

**DOI:** 10.3389/fimmu.2018.01728

**Published:** 2018-07-26

**Authors:** Nicolas Neidert, Alexander von Ehr, Tanja Zöller, Björn Spittau

**Affiliations:** ^1^Department of Molecular Embryology, Faculty of Medicine, Institute for Anatomy and Cell Biology, University of Freiburg, Freiburg, Germany; ^2^Institute of Anatomy, University of Rostock, Rostock, Germany

**Keywords:** Olfml3, microglia, transforming growth factor β1, Smad2, Smad4

## Abstract

Microglia maturation takes place during the postnatal weeks and is characterized by the establishment of a unique microglia-specific gene expression pattern. *Tmem119, Fcrls, Hexb*, and *Olfml3* have been identified among these microglia-specific genes. Transforming growth factor β1 (TGFβ1) has been reported as a critical factor for microglia maturation and maintenance and active TGFβ signaling precedes the inductions of microglial gene expression. In this study, we demonstrate *Olfml3* expression in adult microglia and further provide evidence that TGFβ1 induces upregulation of *Olfml3* expression in postnatal microglia. Using chromatin immunoprecipitation and microglia-specific silencing of TGFβ signaling *in vitro* and *in vivo*, we in clearly show that Olfml3 is a direct TGFβ1/Smad2 target gene. Together, our data underline the importance of TGFβ1 as a critical regulator of microglia functions and microglia maturation and further broaden our understanding of TGFβ1-mediated effects on the resident immune cells of the central nervous system.

## Introduction

Nearly hundred years ago, Pío del Río-Hortega described microglia as the resident immune cells of the central nervous system (CNS) and already proposed a mesodermal origin of these cells in a collection of original papers, which have been made available in translated English versions (1). Novel transgenic approaches have offered a deeper insight into microglia biology and, thus, the microglia origin has just recently been elucidated in detail. In contrast to peripheral macrophages, microglia arise from hematopoietic precursors in the embryonic yolk sac dependent on PU.1 as well as Irf8 signaling pathways (2). During embryonic development, neuron-derived interleukin-34 (IL-34) serves as the most potent factor to guide migrating microglia toward the CNS parenchyma (3). Ginhoux et al. (4) have identified the colony-stimulating factor-1 receptor as being essential to sense neuronal IL-34 and to mediate appropriate microglial colonization of the CNS. Next, to the unique and distinct origin, microglia further adopt a cell type-specific gene expression pattern including Olfactomedin-like 3 (Olfml3), which discriminates these CNS immune cells from other macrophage populations (5–8). The development of this microglia-specific molecular signature occurs during the first postnatal weeks in mice and includes the induction of genes such as *Tmem119, Hexb, Fcrls*, and *Tgfbr1* (9, 10). It is of utmost interest to understand which endogenous factors are involved in the induction of the microglia phenotype and the microglia maturation. However, the detailed molecular mechanisms are not well understood and based on the current knowledge transforming growth factor β1 (TGFβ1) seems to be one of the most important factors. Butovsky et al. (11) have developed TGFβ1-deficient mutant mice, which presented a decrease in postnatal microglia numbers and associated with severe impairment of the establishment of the unique microglia gene expression pattern *in vivo*. Moreover, we have recently described that activated TGFβ signaling precedes the induction of microglia-specific gene expression and further identified the recently introduced microglia marker *Tmem119* as a direct TGFβ1/Smad2 target gene (10). Next, to the regulation of microglia maturation, TGFβ1 has been described as a potent regulator of microglia functions by promoting microglia quiescence (12) and regulating microglia-mediated phagocytosis (13). The fact that TGFβ1 orchestrates postnatal microglia development and further regulates maintenance of adult microglia defines this versatile cytokine as an essential factor for microglia biology. To increase the understanding of TGFβ1-driven microglia maturation, we analyzed the impact of TGFβ signaling on microglial Olfml3 expression. Olfactomedin-like 3 (Olfml3) also referred to as OLF 44 has been introduced as a secreted glycoprotein (14). It is suggested that members of the Olfactomedin-like protein subfamily are involved in the development and functional organization of the CNS and the hematopoietic system (15). In this study, we provide evidence that *Olfml3* expression is restricted to microglia and that TGFβ1 upregulates Olfml3 expression. Using microglia-specific *Tgfbr2*-mutant mice and chromatin immunoprecipitation (ChIP), we further demonstrate that TGFβ signaling is essential for induction of microglial *Olfml3* expression and introduce *Olfml3* as direct TGFβ1/Smad2 target gene. Our data increase the understanding of the molecular mechanisms that regulate microglia maturation and further strengthen the functional importance of TGFβ1 for microglia biology.

## Materials and Methods

### Animals

C57BL/6JRj mice were obtained from Janvier (Le Genest Saint Isle, France) and housed at 22 ± 2°C under a 12 h light/dark cycle with *ad libitum* access to food and water. All animal procedures were conducted in accordance with the German federal animal welfare law, local ethical guidelines of the University Freiburg and have been approved by the animal experimentation committee of the University of Freiburg as well as the Regierungspräsidium Freiburg (G-13/57 [Tgfbr2-MG-KO], X-15/01A [primary microglia]).

### Microglia-Specific Tgfbr2-Knockout Mice

The generation of Cx3cr1^CreERT2^:R26-YFP:Tgfbr2^fl/fl^ mice has been described recently (10). Briefly, mice carrying loxP-site-flanked alleles of *Tgfbr2* were crossed to the Cx3cr1^CreERT2^ mouse line (16). Furthermore, the reporter mouse line B6.129 × 1-Gt(ROSA)26Sortm1(EYFP)Cos/J (17) was introduced to obtain Cx3cr1^CreERT2^:R26-YFP:Tgfbr2^fl/fl^ mice. Cre recombinase activity was induced *in vivo* by treatment of 6- to 8-week-old mice with 8 mg tamoxifen (TAM, T5648, Sigma-Aldrich, Germany) solved in 200 µl corn oil (C8267, Sigma) injected intraperitoneally (two time points 48 h apart). Littermates carrying the respective *loxP*-flanked alleles but lacking expression of Cre recombinase (+/+TAM) or not receiving tamoxifen (cre/+OIL) were used as controls. *In vitro* recombination was achieved using OH-TAM (H7904, Sigma-Aldrich, Germany) at a final concentration of 1 µM to 25 cm^2^ glia culture flasks (single brain cultures) at least 3 days before harvesting microglia. Ethanol (EtOH) was used as a solvent control for *in vitro* experiments.

### Primary Microglia Cultures

Primary microglia cultures were generated as described by Spittau et al. (12). Vessels and meninges were removed from brains of P0/P1 C57BL/6JRj mice (Janvier) and brains were washed and collected in ice-cold Hank’s Buffered Salt Solution (Gibco, Germany). After enzymatic dissociation with Trypsin-EDTA (Gibco, Germany) for 15 min at 37°C, an equal volume of fetal calf serum (FCS, Gibco, Germany) and DNase (Roche, Mannheim, Germany) at a final concentration of 0.05 mg/ml was added. Cells were dissociated using wide- and narrow-bored polished Pasteur pipettes and further centrifuged and resuspended in DMEM/F12 medium (Gibco, Germany) containing 10% FCS and 1% penicillin/streptomycin (Invitrogen). Dissociated cells from two to three brains were plated on poly-d-lysine-coated (Sigma-Aldrich, Schnelldorf, Germany) 75 cm^2^ culture flasks. Cells were kept in a 5% CO_2_/95% humidified atmosphere at 37°C. After 10–14 days in culture, microglia were shaken off (250–300 rpm for 1 h) from adherent astrocytes and plated according to the experimental designs. Treatment with recombinant human TGFβ1 (Peprotech, Hamburg, Germany) was performed at a concentration of 5 ng/ml. For inhibition of microglial TGFβ signaling, a TGFβ receptor type I inhibitor (#616454, Calbiochem, Merck, Darmstadt, Germany) at a final concentration of 500 mM was used. For inhibition of protein biosynthesis, cycloheximide (CHX) (C7698, Sigma-Aldrich, MO, USA) was used at a concentration of 50 µg/ml.

### BV2 Cell Culture

BV2 cells were cultured as recently described by Zhou et al. (18). Briefly, cells were maintained in DMEM/F12 culture medium (Thermo Fisher Scientific) supplemented with 10% FCS and 1% penicillin/streptomycin (Invitrogen) and kept in at 37°C in 5% CO_2_/95% humidified atmosphere. Treatments with 5 ng/ml TGFβ1 (Peprotech, Hamburg, Germany) for ChIP experiments were performed under serum-free conditions.

### RNA Isolation and Reverse Transcription

RNA was isolated from primary microglia using TRIzol reagent (Invitrogen, Karlsruhe, Germany) according to the manufacturer’s instructions. RNA concentration and quality were determined using the NanoDrop 2000 (Thermo Scientific, Germany). 1 µg total RNA from each sample was reverse transcribed to cDNA using Protoscript^®^ II First Strand cDNA Synthesis Kit (#E6560S, New England Biolabs, Frankfurt, Germany) according to the manufacturer’s instructions.

### Quantitative RT-PCR

Quantitative RT-PCR was performed using the CFX Connect™ System (Bio-Rad, München, Germany) in combination with the SYBR Green GoTaq^®^ qPCR Kit (A6002, Promega, Madison, WI, USA). 5 µl of cDNA template was used in 20 µl reaction mixture. Results were analyzed using the CFX Connect™ System (Bio-Rad, München, Germany) Software and the comparative CT method. All data are expressed as 2^−ΔΔCT^ for the gene of interest normalized to the housekeeping gene Gapdh and presented as fold change relative to controls. The following primers have been used throughout this study: Olfml3 *for* 5′-CACCTTGTGGAGTACATGGAAC-3′, Olfml3 *rev* 5′-CTACCTCCCTTTCAAGACGGT-3′ [NM_133859.2], Gapdh *for* 5′-GGCATTGCTCTCAATGACAA-3′, Gapdh *rev* 5′-ATGTAGGCCATGAGGTCCAC-3′ [NM_001289726], Olfml3-SBE1 *for* 5′-TGACAGCTCTAACAGGGCCTA-3′, Olfml3-SBE1 *rev* 5′-ACTCTGACCCCTTGAAAAGGC-3′ [Chr. 3; 103739520-103739508], Olfml3-SBE2 *for* 5′-CCCATCTCTGGTGTCTCTCAC-3′, Olfml3-SBE2 *rev* 5′-TAGTTAAGGCTTCTGGCGACT-3′ [Chr. 3; 103738043-103738055].

### Chromatin Immunoprecipitation

Chromatin immunoprecipitation was performed as recently described (10). The enzymatic ChIP Kit (#9003, Cell Signaling Technology, Inc., Danvers, MA, USA) was used according to the manufacturer’s instructions. After plating of 9 × 10^6^ BV2 cells in 6 × 75 cm^2^ cell culture flasks in DMEM/F12 medium containing 10% FCS and 1% penicillin/streptomycin, BV2 cells were kept 2 h in serum-free medium. Cells were incubated in serum-free medium containing 5 ng/ml TGFβ1. Proteins were cross-linked to the DNA by adding 1% formaldehyde to the cells for 10 min at room temperature (RT). The cells were harvested by scraping them into PBS + Protease inhibitor cocktail. After nuclei preparation with Buffer A and B from the kit, the nuclei from each treatment were separated into four IP samples per treatment. Digestion of chromatin was initiated by adding 0.25 µl Micrococcal Nuclease (#10011, Cell Signaling) for 20 min at 37°C and the nuclei were lysed with three sets of 20 s pulses using a Bioruptor™ (Diagenode, Liège, Belgium) sonicator. Chromatin concentration of every sample was measured using NanoDrop 2000/2000c (Thermo Fisher Scientific, Waltham, MA, USA) and 10 µg of digested, cross-linked chromatin of every sample was used for further steps. A 2% input control was taken aside before starting the IP. A Histone H3 antibody (Cell Signaling, #4620, 10 µl) as a positive control, normal rabbit IgG (Cell Signaling, #2729, 2 µl) as a negative control and Smad2 (Cell Signaling, #5339, 1:100) and Smad4 (Cell Signaling, #38454, 1:100) antibodies were used for IP. The Chromatin was incubated overnight at 4°C. After incubating the chromatin with protein G magnetic beads for 2 h and washing the chromatin in magnetic separation racks (Cell Signaling, #7017), elution of the chromatin was performed. The protein cross-link was reversed by using 2 µl Proteinase K (Cell Signaling, #10012) for 2 h at 65°C. Finally, DNA purification in spin columns was performed and the amount of DNA was quantified by using qPCR. Data are expressed as 2^−ΔCT^ for the Smad-binding element (SBE) of interest normalized to the rabbit IgG control. PCR products were visualized using agarose gel electrophoresis and staining with GelRed (Genaxxon Bioscience). Images were captured using a Biometra (Göttingen, Germany) gel documentation station.

### Immunohistochemistry and Immunocytochemistry

Anesthetized 6-month-old C57BL/6 mice were transcardially perfused using PBS followed by 4% paraformaldehyde (PFA). Afterward, brains were extracted and postfixed in 4% PFA overnight. Free-floating 50 µm thick vibratome (Leica, Wetzlar, Germany) sections were stained overnight with anti-Iba1 (1:500, Wako Chemicals, Japan) and anti-Olfml3 (1:100, sc-243668, Santa Cruz Biotechnology Inc.). Alexa Fluor-488, as well as Alexa Fluor-568-conjugated secondary antibodies (1:200, Cell Signaling Technology) was used for 2 h at RT. Finally, nuclei were stained using 4′6-diamidino-2-phenylindole (Dapi, Roche) for 5 min and sections were mounted on objective slides and covered with Fluoromount G mounting medium. Immunocytochemistry was performed using PFA-fixed primary microglia on glass coverslips as mentioned above. FITC-coupled tomatolectin (Sigma-Aldrich) was used to label microglia and anti-Olfml3 (Santa Cruz Biotechnology Inc.) to detect Olfml3 expression. Localization of Olfml3 in the endoplasmic reticulum (ER) and the Golgi apparatus was confirmed using the Organelle Localization IF Antibody Sampler Kit (Cell Signaling technologies, #8653). Fluorescence-coupled secondary antibodies (1:200, Cell Signaling Technologies) were used for 1 h at RT. Coverslips were washed three times with PBS for 3 min each and nuclei were counterstained using DAPI (Roche). After a final washing (3×), coverslips were mounted on objective slides using Fluoromount G mounting medium (SouthernBiotech). Fluorescence images were captured using the Leica TCS SP8 confocal laser scanning microscope (Leica, Wetzlar, Germany) and the LAS AF image analysis software.

### Statistics

Data are given as mean ± SEM. Statistical differences between two groups were determined using Student’s *t*-test. Multiple-group analysis was performed using one-way ANOVA followed by Bonferroni’s multiple comparison post-test. *P*-values ≤0.05 were considered as statistically significant. All statistical analyses were performed using the GraphPad Prism6 software (GraphPad Software Inc., La Jolla, CA, USA).

## Results

### *Olfml3* Is Expressed in Primary Mouse Microglia and Displays a Cytoplasmic Localization

**Figure 1:**
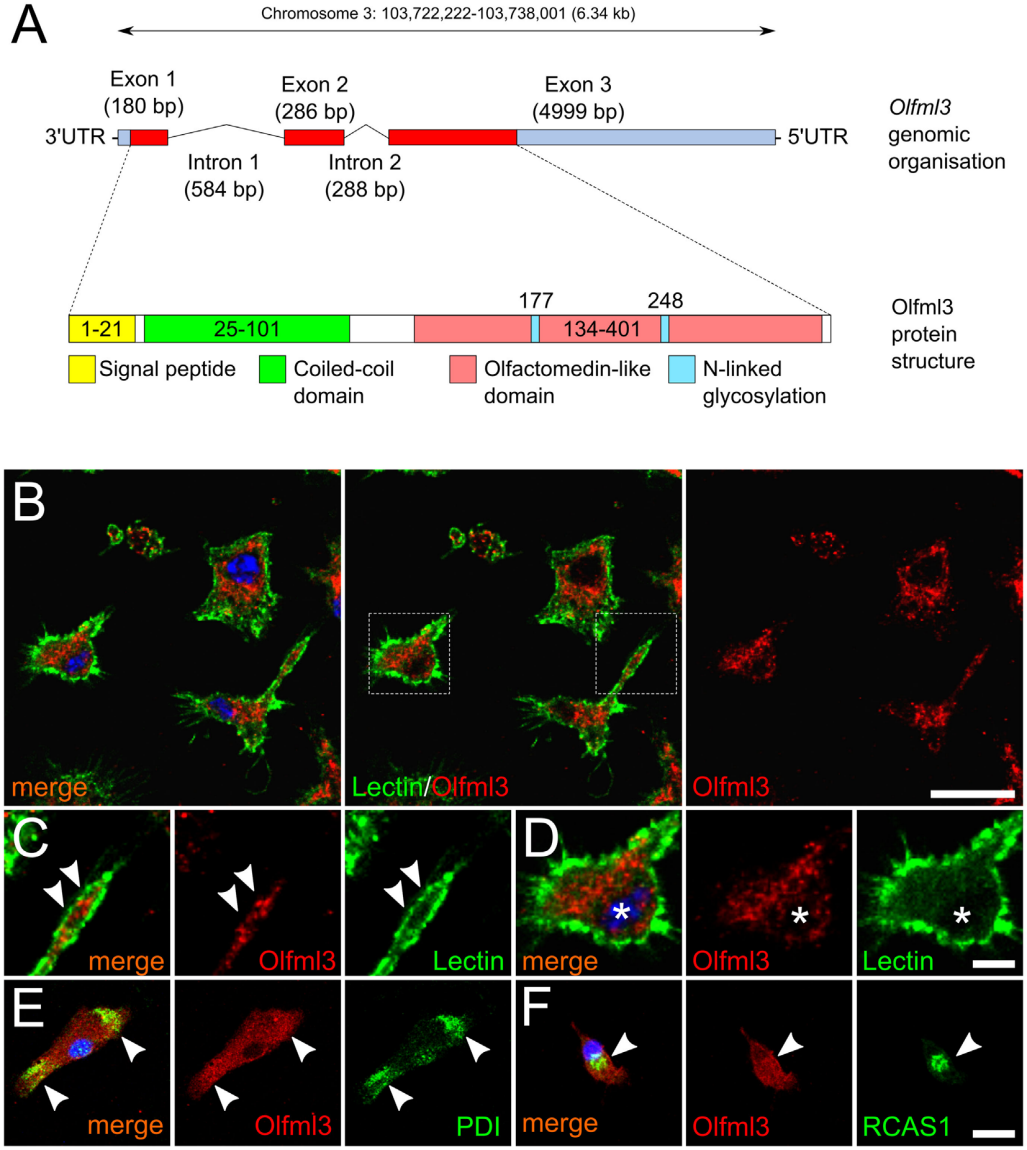
Cytoplasmic localization of Olfml3 in primary microglia. **(A)** Schematic genomic organization and protein structure of Olfml3. **(B)** Immunofluorescence demonstrating the cellular localization of Olfml3 in primary microglia. FITC-coupled tomatolectin was used to label cell membranes. Nuclei were counterstained using DAPI. Dashed rectangles mark areas depicted in panels **(C,D)**. Scale bar indicates 10 µm. **(C)** White arrowheads mark Olfml3 located in microglial processes. **(D)** Olfml3 immunoreactivity in the perinuclear cytoplasm. Asterisks mark the nucleus. Scale bar represents 5 µm. **(E)** Colocalization of Olfml3 with the endoplasmic reticulum (ER) marker protein disulfide isomerase (PDI). White arrowheads mark Olfml3 located in the ER. **(F)** Colocalization of Olfml3 with the Golgi marker RCAS1. White arrowheads mark Olfml3 located in the Golgi apparatus. Scale bar indicates 10 µm.

The Olfml3 gene is located on chromosome 3 and consists of three exons. Figure 1A shows that a 180 bp Exon 1 is separated from Exon 2 (286 bp) by Intron 1, which has a total length of 584 bp. A 288 bp Intron 2 links Exon2 with Exon 3, the latter of which has a length of 4,999 bp and includes a long 5′UTR. The protein structure of Olfml3 as depicted in Figure 1A, demonstrates the presence of 21 aa signal peptide followed by a coiled-coil domain. The central motif is the olfactomedin-like domain which harbors two N-linked glycosylation sites. We further used immunocytochemistry to detect the intracellular localization of Olfml3 in primary microglia. As shown in Figure 1B, Olfml3 immunoreactivity was found in the perinuclear cytoplasm and in microglial processes. FITC-coupled tomatolectin was used to label the cell membrane of primary microglia. Figure 1C displays the presence of Olfml3 speckles in microglial processes suggesting Olfml3 immunoreactivity in exocytotic vesicles. The perinuclear staining pattern of Olfml3 suggests an ER localization (Figure 1D). To confirm this subcellular localization, protein disulfide isomerase (PDI) and receptor binding cancer antigen expressed on SiSo cells (RCAS1) were used as markers for the ER and the Golgi apparatus, respectively. As shown in Figure 1E, Olfml3 colocalized with PDI indicating presence in the ER. Moreover, Olfml3 further displayed accumulation in the Golgi apparatus as evidenced by colocalization with RCAS1 (Figure 1F). Together, these data demonstrate that primary microglia show robust expression of the secreted glycoprotein Olfml3.

### Olfml3 Is Expressed in Microglia *In Vivo*

**Figure 2:**
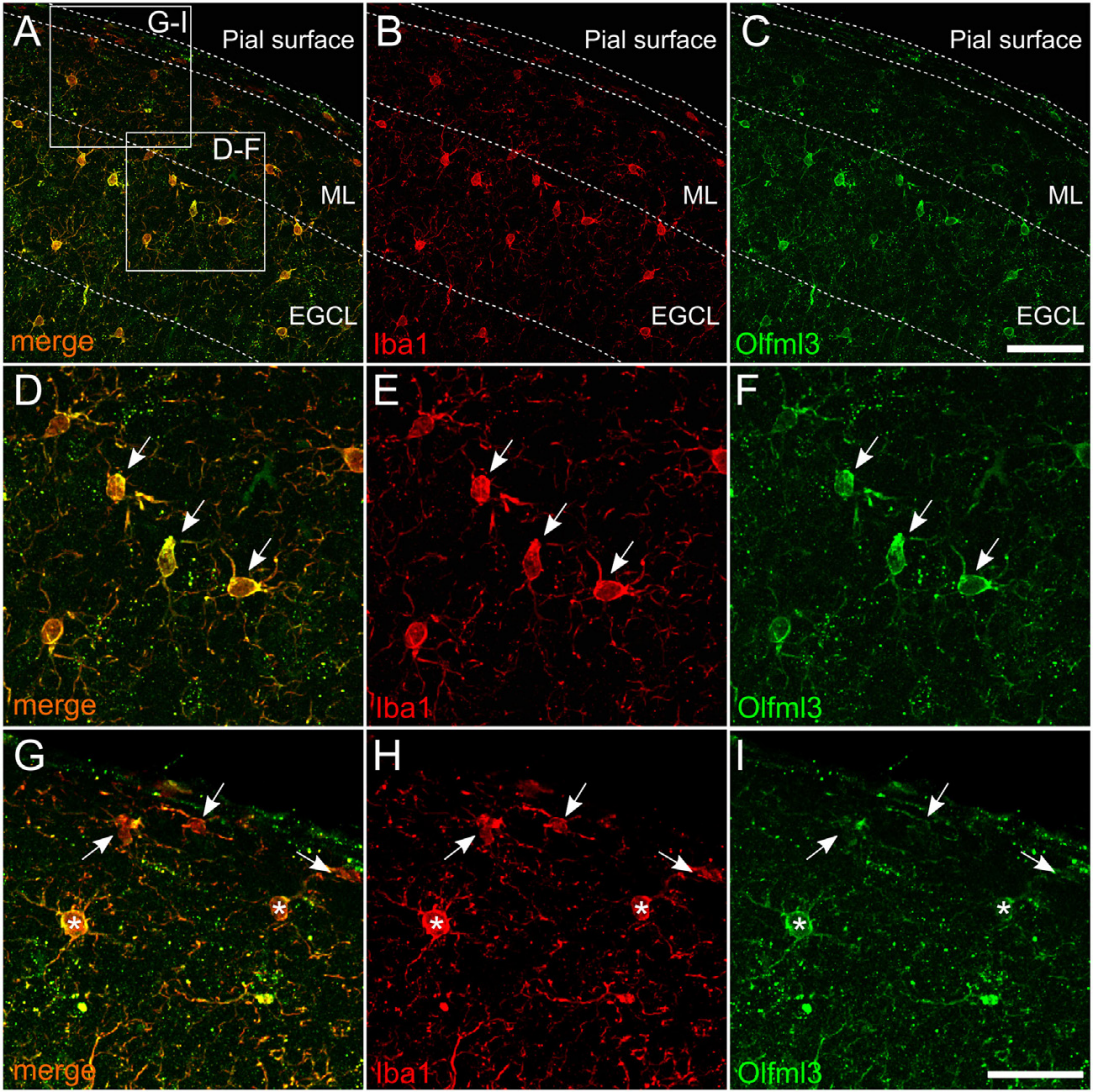
Expression of *Olfml3* in cortical microglia. Overlay **(A)** of Iba1^+^ **(B)** and Olfml3^+^ **(C)** cortical microglia from 6-month-old C57BL/6 mice demonstrate microglia-specific expression of Olfml3. White arrows in high magnification images show strong cytoplasmic immunoreactivity for Olfml3 **(F)** in Iba1^+^ microglia **(D,E)**. Noteworthy, Iba1^+^ meningeal macrophages (white arrows) located in close proximity to the pial surface show faint Olfml3 expression as compared to microglia (white asterisks) of the ML **(G–I)**. Abbreviations: ML, molecular layer; EGCL, external granule cell layer. Scale bars represent 20 µm **(A–C)** and 10 µm **(D–I)**.

It has been described that Olfml3 belongs to a set of genes, which have been demonstrated to be microglia-specific and, which are not expressed by other macrophage populations (5, 6, 8, 9). Since these data were obtained from RNAseq and/or gene expression studies, we used immunohistochemistry to determine Olfml3 expression in cortical microglia of adult mice. As shown in Figure 2, Olfml3 immunoreactivity could be detected in the molecular layer and the external granule cell layer of the frontal cortex (Figure 2A). Iba1^+^ cortical microglia (Figure 2B) were also positive for Olfml3 (Figure 2C). High magnification images reveal that strong Olfml3 immunoreactivity was detectable in the cytoplasm of Iba1^+^ microglia (Figures 2D–F). Interestingly, microglial processes only displayed weak Olfml3 signals and Olfml3 speckles could be found in the periphery of microglia suggesting an extracellular localization and accumulation (Figure 2F). Iba1^+^ meningeal macrophages can be found in close proximity to the pial surface and, thus, Olfml3 immunoreactivty in this non-microglial cell was analyzed. As depicted in Figures 2G–I, Iba1^+^ meningeal macrophages (white arrows) showed no or very faint Olfml3 immunoreactivity whereas Iba1^+^ cortical microglia (white asterisks) show strong Olfml3 expression. These data clearly show that Olfml3 is predominantly expressed by Iba1^+^ microglia and further suggest that Olfml3 is secreted and accumulated in the extracellular space.

### Microglial Olfml3 Expression Is Dependent on TGFβ Signaling

**Figure 3:**
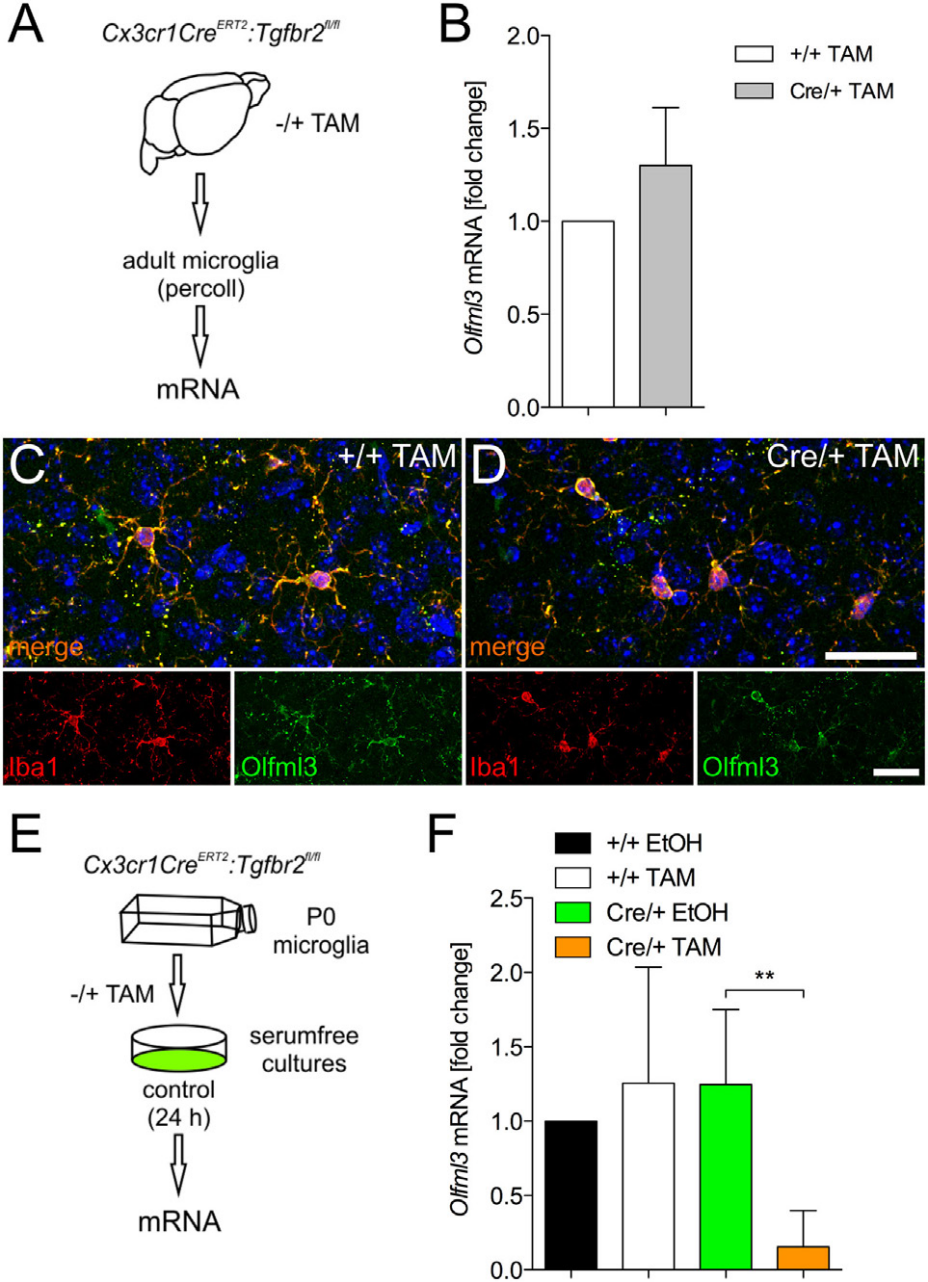
Identification of *Olfml3* as a transforming growth factor β1 (TGFβ1)-regulated gene. **(A)** Scheme illustrating the process of mRNA isolation from adult microglia of *Cx3cr1^CreERT2^:Tgfbr2^fl/fl^* transgenic mutant mice. **(B)** Expression of *Olfml3* in adult wild-type (+/+TAM) and *Tgfbr2* mutant microglia (Cre/+TAM). Immunohistochemistry to monitor expression of Olfml3 in cortical microglia of wild-type (+/+TAM) **(C)** and *Tgfbr2*-deficient (Cre/+TAM) mice **(D)**. Scale bars indicate 10 µm. **(E)** Scheme summarizing TAM-induced recombination and isolation of postnatal microglia *in vitro* isolated from P0/P1 mice. **(F)** Significantly decreased expression of *Olfml3* in *Tgfbr2*-deficient (Cre/+TAM) postnatal microglia recombined *in vitro*. Data are presented as mean ± SEM from at least three independent experiments **(B,F)**. *P*-value derived from one-way ANOVA is **P < 0.01.

It has recently been described that TGFβ1 is necessary to induce the expression of microglia-specific genes such as *Olfml3* (11). Moreover, we have demonstrated that microglial TGFβ signaling at P7 precedes the upregulation of microglia-specific genes *in vivo* (10). To address whether the Olfml3 expression in microglia is dependent on TGFβ signaling, we used microglia from *Cx3cr1^CreERT2^:Tgfbr2^fl/fl^* mice. As displayed in Figure 3A, 2-month-old *Cx3cr1^CreERT2^:Tgfbr2^fl/fl^* mice were used to generate the tamoxifen-induced microglia-specific deletion of ligand-binding receptor *Tgfbr2 in vivo*. Four weeks after recombination, microglial RNA was isolated and used to detect Olfml3 expression. Figure 3B shows that silencing of TGFβ signaling in adult microglia did not impair Olfml3 expression. Moreover, normal Olfml3 expression was observed in cortical Iba1^+^ microglia after deletion of *Tgfbr2 in vivo* (Figure 3D) when compared with wild-type microglia (Figure 3C). Using postnatal microglia cultures from *Cx3cr1^CreERT2^:Tgfbr2^fl/fl^* mice and tamoxifen-induced recombination *in vitro* (Figure 3E), we observed that Olfml3 expression was significantly reduced in *Tgfbr2*-deficient microglia (Figure 3F). In summary, the presented data indicate that TGFβ signaling is essential to induce Olfml3 expression in immature postnatal microglia but is dispensable to maintain Olfml3 expression in mature adult microglia. The results further suggest that Olfml3 might be a direct TGFβ1 target gene.

### Identification of *Olfml3* as a Direct TGFβ1/Smad2 Target Gene

**Figure 4:**
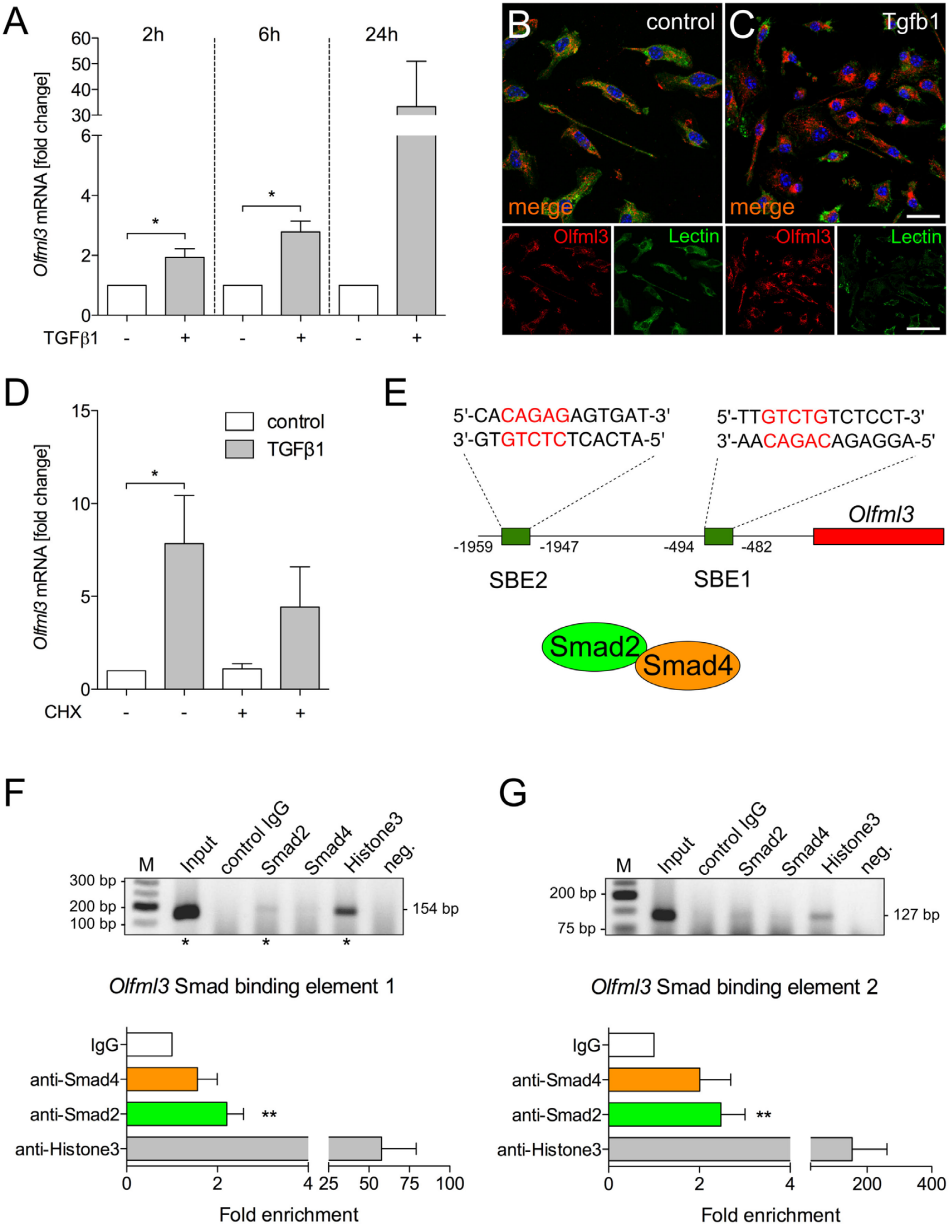
Identification of *Olfml3* as a direct transforming growth factor β1 (TGFβ1)/Smad2 target gene **(A)** Increased expression of *Olfml3* in primary microglia treated with recombinant TGFβ1 (5 ng/ml) for indicated time points. Immunocytochemistry demonstrating expression of Olfml3 in control **(B)** and TGF treated primary microglia **(C)** after 24 h. FITC-coupled tomatolectin was used to label microglia. Scale bars represent 10 µm for merged images and 20 µm for single stain images. **(D)** Cycloheximide-induced inhibition of protein synthesis only marginally affected TGFβ1-induced expression of *Olfml3* in primary microglia. **(E)** Genomic organization of *Olfml3* displaying localization and sequence of *in silico*-predicted Smad-binding elements (SBEs). Results of chromatin immunoprecipitation (ChIP) PCR amplification and quantifications of qPCR results after ChIP of TGFβ1-treated BV2 microglia using Smad4- and Smad2-specific antibodies. Significant enrichment after Smad2 precipitation was detected for SBE1 **(F)** as well as for SBE2 **(G)**. Asterisks mark Olfml3 promoter PCR product separated by agarose gel electrophoresis **(F,G)**. Smad4 binding could not be observed for all SBEs. Anti-Histone3 antibodies were used as a positive control and non-specific isotype IgGs were used as a negative control for all ChIP experiments. Data are presented as mean ± SEM from at least three independent experiments. *P*-values derived from Student’s *t*-test are *P < 0.05 **(A,F,G)**. *P*-values derived from one-way ANOVA are *P < 0.05 **(D)**.

To address whether *Olfml3* is direct TGFβ1 target gene, primary microglia were treated with recombinant human TGFβ1 (5 ng/ml) for 2, 6, and 24 h. As depicted in Figure 4A, significant upregulation of Olfml3 expression was detected after 2 and 6 h. Although Olfml3 expression was increased after 24 h of TGFβ1 treatment, no significant changes were detectable. Next, immunocytochemistry was employed to monitor increases in Olfml3 expression in primary microglia. Whereas a distinct but weak immunoreactivity for Olfml3 could be observed in untreated microglia (Figure 4B) strong increases in Olfml3 signals were detectable after treatment of primary microglia with TGFβ1 for 24 h (Figure 4C). Using CHX-mediated inhibition of protein synthesis revealed that TGFβ1-induced Olfml3 expression was independent on *de novo* synthesis of proteins (Figure 4D) indicating a direct upregulation of Olfml3 transcription by activated TGFβ signaling. *In silico* analysis of the Olfml3 promoter region revealed the presence of two putative SBEs upstream of the transcriptional start site. Both SBEs contain the typical palindromic CAGAC DNA-binding sequence for Smads (Figure 4E). BV2 cells were treated with TGFβ1 for 2 h and ChIP was performed using Smad2- as well as Smad4-specific antibodies. Figures 4F,G show that Smad2 interacted with both putative SBEs whereas Smad4 did not. Precipitation of Histone3 was used as the positive control for all performed ChIP experiments. These data clearly demonstrate that Olfml3 is a direct TGFβ1/Smad2 target in microglia and further show that Smad4 is not interacting with SBEs of the Olfml3 promoter.

## Discussion

In this study, we have demonstrated that TGFβ1 induces the expression of the microglia-specific gene *Olfml3* in primary microglia. Moreover, we provide evidence that *Olfml3* is a direct TGFβ1/Smad2 target gene and further revealed that postnatal microglial TGFβ signaling is essential for Olfml3 upregulation whereas lack of TGFβ1 signal transduction is dispensable for the maintenance of microglial Olfml3 expression *in vivo*.

We have recently demonstrated that microglial TGFβ signaling is activated at postnatal day 7 (P7) and, thus, precedes the establishment of the microglial gene expression pattern (10). Using ChIP, we identified Olfml3 as a direct Smad2 target gene. Interestingly, Smad4 could not be demonstrated to sufficiently bind the SBEs of the Olfml3 promoter. This observation is in congruence with the recently reported activation of *Tmem119* expression in microglia (10) and raises the question to which extent Smad4 is necessary for TGFβ1-mediated regulation of microglial gene expression. The canonical TGFβ signaling pathway has been described to involve the TGFβ-induced formation of hetero-oligomeric complexes of Tgfbr2 and Tgfbr1 (19) followed by Tgfbr1-induced phosphorylation of Smad2 at serine residues 465 and 467, which is the prerequisite for Smad2–Smad4 complex formation and subsequent nuclear translocation (20). In this study, Smad4 was not involved in Olfml3 expression and it remains to be established whether Smad4 is needed for TGFβ1-induced effects in microglia. It has been described that Smad2 is able to directly interact with nucleoporins Nup214 and Nup153 to undergo Smad4-independent nuclear translocation (21). This displays a potential mechanism to explain the observed Smad2-dependent regulation of *Olfml3* expression in microglia. However, further sophisticated studies are necessary to address the Smad2-dependent and Smad4-independent TGFβ1 effects in microglia.

Using microglia-specific deletion of microglial Tgfbr2, we demonstrated that postnatal microglia showed a significant reduction in *Olfml3* expression, which further supports the notion that TGFβ1 is a critical factor to induce maturation of microglia at early postnatal stages. Noteworthy, this reduction in *Olfml3* expression was not detectable when TGFβ1 signaling was abrogated in adult microglia *in vivo*. Here, normal microglial *Olfml3* expression was observed in mutant mice indicating that intact TGFβ signaling is dispensable for maintenance of *Olfml3* expression. Most studies reporting microglia-specific *Olfml3* expression have used mRNA-based expression data (8, 9, 11) and, thus, the protein distribution of Olfml3 was unknown. Here, we present data demonstrating cytoplasmic Olfml3 protein localization *in vitro* and *in vivo*. Moreover, the observed speckled *in vivo* staining pattern (Figure 2) suggests the extracellular accumulation of secreted Olfml3. It remains unclear what physiological functions are linked to microglia-derived Olfml3 *in vivo*. In this study, we have further demonstrated that Olfml3 expression in the CNS is restricted to microglia, whereas meningeal macrophages and other neural cells including neurons, astroglia, and oligodendroglia did not show Olfml3 expression. However, abundant mRNA levels of human OLFML3 (hOLF44) have been described to be detectable in the placenta, liver, and heart (14). Moreover, genetic disruption of *Olfml3* in mice using a LacZ-knock-in strategy further revealed expression of Olfml3 during early embryogenesis in the allantois, lateral plate mesoderm as well as in the heart and the CNS. Interestingly, the homozygous mutant mice were viable and fertile and no obvious morphological phenotype has been reported (22). Nevertheless, the structural similarity to other olfactomedin proteins implies also related functions of Olfml3. The conserved olfactomedin-like domain, which is thought to mediate protein-protein interactions, has been described in organisms ranging from nematodes to humans (23). For instance, zebrafish olfactomedin 1 (Olfm1) regulates retinal axon elongation (24) by interacting with the Nogo A receptor and, thus, preventing the collapse of the growth cone (25). Furthermore, deletion of Olfm1 in mice resulted in structural and functional impairments of white matter tracts and the olfactory system (26). In contrast to Olfm1, genetic targeting of Olfm2 did not result in gross abnormalities but mutant mice presented with abnormal locomotor coordination, reduced exploration and anxiety-related behavior (27). These studies underline the importance of olfactomedins for neurodevelopment and neuronal functions and suggest a contribution of Olfml3 in developmental and functional aspects of the postnatal CNS. Taken together, our results introduce Olfml3 as a new TGFβ1/Smad2 target gene in microglia and, thereby, broaden our understanding of TGFβ1-mediated regulations of microglia maturation. The role of microglia-derived Olfml3 remains elusive and further studies are necessary to elucidate the importance of microglia-derived Olfml3.

## Ethics Statement

This study was carried out in accordance with the recommendations of the German federal animal welfare law and local ethical guidelines of the University Freiburg. The protocol was approved by the Regierungspräsidium Freiburg.

## Author Contributions

BS conceived the project and wrote the manuscript. NN, AE, TZ, and BS performed experiments and analyzed the data. All the authors have read and approved the final manuscript and further agreed to be accountable for the content of the work.

## Conflict of Interest Statement

The authors declare that the research was conducted in the absence of any commercial or financial relationships that could be construed as a potential conflict of interest.
